# IMPACT OF COMMUNICATION ON INTERACTIONS BETWEEN FAMILY CAREGIVERS AND HEALTH CARE PROFESSIONALS

**DOI:** 10.1093/geroni/igae098.3390

**Published:** 2024-12-31

**Authors:** Nadia Khan, Donna Benton, Yujun Zhu

**Affiliations:** University of Southern California Leonard Davis School of Gerontology, Los Angeles, California, United States; University of Southern California, Los Angeles, California, United States; University of Southern California, Los Angeles, California, United States

## Abstract

Family members providing care to older adults with progressive conditions are at an increased risk for caregiver burnout and poorer health. Few studies have examined how healthcare professionals interact with and support the family caregivers of their patients. This study investigated the perceived attitudes of healthcare professionals toward family caregivers and the experiences of family caregivers in healthcare settings where their loved ones receive medical treatment. We used a voluntary, anonymous survey through the software REDCap composed of Likert-scale questions to obtain the opinions of family caregivers to older adults diagnosed with a memory impairment (N=199). We conducted linear regression to examine the relationship between demographic characteristics and the quality of communication between caregivers and healthcare providers. Results showed that 57% of participants strongly disagreed or disagreed that their healthcare team gave them information on how to care for themselves while caring for loved ones. Preliminary linear regression analysis showed that there is a statistically significant positive relationship (p< 0.05) between the discussion of caregiver self-care and the demographic variables of income and education level. Additionally, 48% of participants strongly disagreed or disagreed that their healthcare team asked them about their well-being and quality of life. Our initial findings have implications for how healthcare professionals communicate with family caregivers about their well-being and the emotional burden of family caregiving. Additionally, our results demonstrate the need for new communication strategies between healthcare professionals and family caregivers that will effectively address their diverse needs. Word Count: 244

